# Pasture utilization, behavior, and egg quality of Chilean creole and Lohmann brown hens in a rotational free-range system^☆^

**DOI:** 10.1016/j.psj.2026.107351

**Published:** 2026-06-26

**Authors:** Mónica Gandarillas, Catalina Guarda, Juan Pablo Keim, Iván Calvache, Ángel Hernández, Christian Guillermo Chávez, Tamara Tadich

**Affiliations:** a Instituto de Producción Animal. Facultad de Ciencias Agrarias y Alimentarias. Universidad Austral de Chile. Independencia 631. Valdivia 5110566, Chile; b Instituto de Ciencia Animal. Facultad de Ciencias Veterinarias. Universidad Austral de Chile, Chile; c Escuela de Graduados, Facultad de Ciencias Agrarias y Alimentarias, Universidad Austral de Chile, Chile

**Keywords:** Free-range system, Chilean creole hens, Grazing behavior, Egg quality, Yolk pigmentation

## Abstract

Free-range systems integrate pasture access with laying hen production; however, genotype-specific responses under rotational grazing remain insufficiently characterized, particularly for local genetic resources. This study evaluated grazing behavior, pasture utilization, egg production, egg quality traits, and functional egg components of Chilean Creole (ChC) and Lohmann Brown (LB) hens managed in a rotational free-range system in southern Chile. Thirty hens (70 weeks of age) were assigned to two genotypes with three replicate plots per genotype and five hens per plot in a completely randomized design with repeated measures over 91 days. A total of 551 eggs (260 LB; 291 ChC) were evaluated for external and internal quality traits. Behavioral time budgets, sward characteristics, and apparent dry matter intake were recorded, and fatty acids and vitamins A and E were analyzed descriptively using composite yolk samples. Chilean Creole hens showed greater apparent forage intake than Lohmann Brown hens, with a significant genotype × period interaction (*P* < 0.01), whereas egg production and egg weight did not differ between genotypes (*P* > 0.05). Chilean Creole eggs exhibited greater yolk pigmentation and a higher shell proportion than Lohmann Brown eggs (*P* < 0.05), while Lohmann Brown eggs had greater fresh shell weight (*P* < 0.001). Under controlled rotational management with concentrate supplementation, genotype affected apparent forage intake and selected egg quality traits, whereas yolk fatty acids and vitamins provided exploratory evidence of changes associated with pasture access.

## Introduction

Free-range (FR) egg production systems provide an opportunity to integrate animal welfare, pasture use, and product differentiation in laying hen production. By providing outdoor access, these systems allow hens to express a broader range of natural behaviors, including perching, nesting, dust bathing, wing flapping, and foraging (Sossidou et al., 2011). In addition to their welfare implications, FR systems expose hens to a variable nutritional environment derived from herbage, seeds, and invertebrates, which may influence both productive responses and egg quality traits (Karsten et al., 2010).

Pasture intake can contribute bioactive compounds such as carotenoids, tocopherols, and polyunsaturated fatty acids (PUFA), with potential effects on yolk pigmentation, fatty acid composition, and the omega-6:omega-3 ratio of eggs (Karsten et al., 2010; Singh and Cowieson, 2013). However, these responses are not always consistent because pasture use and forage intake vary substantially among hens and are influenced by management, environmental conditions, and individual ranging behavior (Chielo et al., 2016; Wurtz et al., 2023). Moreover, pasture condition itself changes over time, and hens may concentrate their activity near shelters, causing uneven pasture use, soil disturbance, and sward degradation (Campbell et al., 2017; Pettersson et al., 2016). Therefore, studies conducted under FR conditions should integrate pasture measurements with behavioral, productive, and egg-quality outcomes to better interpret bird responses.

Genotype is a key factor that may modulate the use of outdoor resources and the productive efficiency of hens in FR systems (Mugnai et al., 2009). Genotype × environment interactions have been reported for laying performance and egg quality traits under different housing conditions (Lordelo et al., 2017; Rakonjac et al., 2021), and recent evidence continues to show genotype-dependent responses in performance and egg quality under FR management (Aygun et al., 2025). Nevertheless, most studies have focused on commercial laying strains, particularly brown-egg hybrids, while local genetic resources have received less attention. This limits the ability to determine whether locally adapted hens differ from commercial hybrids in foraging behavior, pasture utilization, and egg quality responses under pasture-based systems.

This gap is particularly relevant in Chile, where the Chilean Creole (ChC) hen represents a local genetic resource associated with family-based and low-input poultry systems (Asencio et al., 2023). Its rusticity and potential adaptation to outdoor conditions suggest that it may be suitable for pasture-based egg production. However, quantitative comparisons between ChC hens and commercial hybrids under standardized FR conditions remain scarce, especially when pasture characteristics, grazing behavior, productive performance, egg physical quality, and yolk functional components are evaluated simultaneously. Such information is important because egg quality traits influenced by diet and environment, such as yolk color, are also relevant for consumer perception and market differentiation (Berkhoff et al., 2020).

Previous research under Chilean FR conditions has shown that commercial laying genotypes may differ in pasture consumption, egg weight, yolk pigmentation, and albumen quality (Gandarillas et al., 2025). However, evidence for local genetic resources such as ChC hens, particularly under rotational pasture management, remains limited. The present study addresses this gap by comparing ChC hens with Lohmann Brown hens in a rotational FR system in southern Chile, integrating pasture utilization, behavioral patterns, egg production, egg quality, and selected yolk functional components.

The objective of this study was to compare pasture utilization, behavioral patterns, egg production, egg quality traits, and selected yolk functional components of Chilean Creole and Lohmann Brown hens managed in a rotational FR system in southern Chile. We hypothesized that Chilean Creole hens would exhibit greater pasture utilization and foraging activity than Lohmann Brown hens while maintaining comparable egg production and egg weight. Yolk fatty acids and vitamins A and E were evaluated as exploratory outcomes because composite yolk samples did not allow inferential statistical analysis.

## Materials and methods

### Ethical approval

All experimental procedures were approved by the Institutional Animal Care and Use Committee (CICUA) of Universidad Austral de Chile (approval No. 527/2023) and were conducted in accordance with Chilean Law No. 20.380 on Animal Protection.

### Location and experimental period

The study was conducted at the Austral Agricultural Research Station (EEAA), Universidad Austral de Chile, Valdivia, Chile (39°48′S, 73°15′W). The experimental period lasted 91 days, from November 1, 2023, to January 31, 2024. Before the beginning of the experiment, hens had been maintained under a floor-housing system and had no previous experience with rotational free-range management or direct pasture use. Thus, a two-week acclimation period was conducted prior to the experimental phase to allow birds to adapt to the free-range environment and housing conditions.

External and internal egg quality analyses were performed at the Animal Nutrition Laboratory of the Instituto de Producción Animal, Universidad Austral de Chile.

### Experimental design and birds

The experiment was arranged as a completely randomized design with repeated measures over time. The fixed treatment factor was genotype, with 2 levels: Chilean Creole and Lohmann Brown. A total of 30 laying hens, approximately 70 wk of age at the beginning of the experiment, were used. Fifteen hens per genotype were allocated to 3 replicate plots per genotype, with 5 hens per plot. The plot was considered the experimental unit for pasture measurements, apparent pasture intake, behavioral observations, egg production, and egg quality traits.

The experimental period lasted 91 d, from November 1, 2023, to January 31, 2024, after a 2-wk adaptation period. Pasture variables were evaluated during 5 consecutive grazing periods associated with the rotational movement of plots. Egg quality traits were evaluated on predefined sampling dates across the experimental period and averaged by plot before statistical analysis.

### Housing and free-range management

Outdoor stocking density was one hen per 4 m², complying with the EU free-range egg production standard, within the broader framework of European laying hen welfare regulations (Council of the European Union, 1999; European Commission, 2008).

Each plot housed five hens and was equipped to meet EU welfare requirements. Housing consisted of a mobile shelter providing overnight protection against predators and adverse weather. Perches were installed providing a minimum of 15 cm of linear perch space per hen. Each plot included one nest box, exceeding the minimum requirement of one nest per seven hens. A dust-bathing area containing dry substrate was provided to allow natural behavior expression.

Feed was supplied in a circular feeder allowing simultaneous access, and water was provided ad libitum using a bell drinker system. A 90 percent shade mesh was installed in each plot to provide thermal comfort and protection from direct solar radiation.

Plots were relocated every 10 days to maintain vegetation cover and prevent pasture degradation. Birds were introduced into plots when pasture height ranged between 10 and 15 cm and were moved once residual height declined after grazing.

### Feeding management

Hens received 120 g/hen per day of a balanced concentrate formulated for laying hens. The diet consisted of 60% ground corn, 24% soybean meal, 5% wheat middlings, 8.5% calcium carbonate, 1.4% dicalcium phosphate, 0.35% salt, and a vitamin-mineral premix (Veterquímica®, Chile).

### Pasture preparation and management

Prior to the experimental period, pasture was homogenized using a lawn mower (Husqvarna LC151), leaving a residual height of approximately 15 cm. Homogenization cuts were performed on September 30 and October 25, 2023. Fertilization was applied on October 2, 2023, using 25 kg of urea for pasture maintenance.

Pasture consisted of naturalized polyphytic swards including *Holcus lanatus, Bromus valdivianus, Lolium perenne, Dactylis glomerata, Anthoxanthum odoratum, Trifolium repens, Trifolium pratense, Plantago lanceolata, Hypochaeris acaulis, and Taraxacum officinale.*

### Pasture measurements

Pasture height was measured before bird entry (target 10–15 cm and full vegetative cover) and after each 10-day grazing period. Seven random measurements per plot were taken to determine residual height.

Botanical composition was assessed using a 20 × 20 cm metal quadrat placed randomly within each plot. Two samples per plot were collected at ground level before and after grazing. Samples were separated by species, oven-dried at 60°C for 48 hours, and expressed on a dry matter basis. Species were grouped into grasses, legumes, and broadleaf species. Differences between pre- and post-grazing composition were used to estimate plant preference.

### Herbage availability and dry matter

Herbage availability was determined by clipping forage within a 20 × 20 cm quadrat at ground level. Fresh weight was recorded using a digital scale (Model DH 2000, Quimis, Diadema, SP, Brazil). Samples were dried at 60°C for 72 hours to determine dry matter content. Apparent dry matter intake was calculated as the difference between pre- and post-grazing dry matter yield divided by the number of hens and grazing days per plot. Nitrogen concentration was determined using a Leco FP-428 Nitrogen Determinator (Leco Corporation, USA), and crude protein was calculated as nitrogen multiplied by 6.25.

### Egg production

Egg production was recorded daily for 60 consecutive days. All eggs were counted and weighed to determine egg production and egg weight per plot.

### Egg quality assessment

External and internal egg quality were evaluated throughout the experimental period. A total of 551 eggs (260 Lohmann Brown; 291 Chilean Creole) collected on predefined sampling days across the experimental period were evaluated for external and internal quality traits. Data were averaged per plot before statistical analysis.

### External egg quality

Eggs were collected daily between 10:00 and 11:00 h, weighed, and classified according to Chilean Standard 1376 Of78 (Instituto Nacional de Normalización, 1978) into the following categories: Special (>68.0 g), Extra Large (61.1–68.0 g), Large (54.1–61.0 g), Medium (47.1–54.0 g), Small (40.1–47.0 g), and Very Small (<40.0 g). Egg weight (EW) was determined using an electronic balance (Q-DG2000, Quimis®, São Paulo, Brazil; ±0.05 g). Egg length, measured from pole to pole, and egg width, measured at the equatorial diameter, were determined using a digital caliper. Egg shape index was calculated as ESI = (egg width / egg length) × 100. Degree of shell dirtiness was visually evaluated using a 5-point scale, where 1 = clean shell, 2 = slightly dirty, 3 = moderately dirty, 4 = very dirty, and 5 = completely dirty. Shells were carefully separated from albumen and membranes, oven-dried at 60°C for 48 h, and weighed. Shell percentage was calculated as (dry shell weight / egg weight) × 100. Shell thickness was measured at three points, including the air cell, equator, and sharp end, using a digital caliper, and the average value was recorded.

### Internal egg quality

Eggs were broken individually onto a flat glass surface for internal quality evaluation. Albumen quality was assessed by measuring thick albumen height (AH) using a digital caliper. Haugh units (HU) were calculated using the standard formula: HU=100 × log_10_ (h − 1.7w^0.37^+7.6) where h is albumen height (mm) and w is egg weight (g).

Yolk color was assessed using the DSM YolkFan™ color scale (DSM, 2021), ranging from 1 (pale yellow) to 16 (dark orange). Afterwards, yolks were carefully separated from albumen and weighed to determine yolk weight (YW), while albumen weight (AW) was calculated by difference. Yolk height (YH) was measured using a digital caliper. Yolk ratio (YR, %) and albumen ratio (AR, %) were calculated as YR = (YW / EW) × 100 and AR = (AW / EW) × 100, where EW represents egg weight.

### Functional components: fatty acids and vitamins

Egg yolk samples were collected at four time points including baseline and during grazing. Composite samples were prepared per treatment by pooling yolks from the three replicate plots. Samples were frozen, lyophilized (Virtis Benchtop K, USA), and analyzed at the Institute of Nutrition and Food Technology (INTA), University of Chile. Fatty acid profile was determined by gas chromatography with flame ionization detection (GC-FID) according to AOAC Method 991.39 and AOCS Official Method Ce 1b-89. Vitamin E was determined by high-performance liquid chromatography with diode array detection (HPLC-DAD). Vitamin A was determined by high-performance liquid chromatography with ultraviolet detection (HPLC-UV). Because composite samples were used at the treatment level, functional nutrient results are presented descriptively and were not subjected to inferential statistical analysis.

### Behavioral observations

Behavioral observations were conducted to quantify time budget allocation and range use in Lohmann Brown (LB) and Chilean Creole (ChC) hens under the rotational free-range system described previously.

Observations were carried out between 12:00 and 14:00 h, corresponding to the period of highest daytime activity, when hens had access to the outdoor range. Observations were performed on five sampling days distributed throughout the experimental period.

Due to the high variability and unpredictability of individual behaviors, a scan sampling method was applied following Martin and Bateson (2007). During each scan, all visible birds within each plot were simultaneously observed, and the number of hens expressing each behavioral category was recorded. Each observation cycle lasted 6 minutes, consisting of 3 minutes of acclimation, during which the observer remained stationary to minimize disturbance, followed by 3 minutes of behavioral recording. The 3-minute period was divided into three 1-minute sampling units, and the number of hens that expressed each behavior within that minute was recorded, and an average was obtained for the 3-minute period. All observations were conducted by a single trained observer, and each pen observation at a given time point was considered one replicate for statistical analysis.

During each scan, hens were categorized into one of the mutually exclusive behavioral states described in the ethogram (Table 1). Behavioral data were expressed as the percentage of total observations allocated to each behavioral category per plot per observation day.

**Table 1:** Description of the mutually exclusive behaviors observed.

Behavioral category	Behavior	Description
Inactivity behavior	Sleeping	Resting with eyes closed, in a crouched or standing position without activity.
Comfort behaviours	Preening	Grooming own feathers using the beak.
Comfort behaviours	Head scratching	Scratching the head or neck area with a foot.
Active behaviours	Locomotion	Moving from one point to another, usually walking, without active foraging or social interaction.
Active behaviours	Feeder pecking	Pecking at or around the feeder without active feed consumption.
Agonistic behaviours	Confrontation	Aggressive interactions directed toward another hen.
Agonistic behaviours	Feather pecking	Directing pecks at the feathers of another hen.
Ingestive behaviours	Eating	Consuming feed from the feeder.
Ingestive behaviours	Foraging	Pecking at vegetation, scratching soil, or ingesting plant material or invertebrates in the outdoor range.
Ingestive behaviours	Drinking	Consuming water from the drinker.

### Statistical analyses

The plot was used as the experimental unit for all inferential analyses. Data for pasture characteristics, apparent pasture intake, behavioral traits, egg production, and egg quality were averaged by plot before analysis. Genotype was considered the main fixed effect. Time was included as a repeated factor when variables were measured repeatedly during the experimental period.

For pasture characteristics and apparent pasture intake, time corresponded to five consecutive grazing periods associated with the rotational use of pasture plots. For egg quality traits, time corresponded to the same five grazing periods, plus an additional baseline sampling period at the start of the trial, designated as P0. For behavioral traits, time corresponded to observation days distributed throughout the experiment.

Repeated-measures analyses were performed using the MIXED procedure of SAS version 9.4 (SAS Institute Inc., Cary, NC). The model included genotype, time, and the genotype × time interaction as fixed effects. Plot nested within genotype was used as the subject of the repeated statement. The residual covariance structure was selected according to Akaike’s information criterion. Least squares means were compared using Tukey adjustment when significant effects were detected. Statistical significance was declared at *P* ≤ 0.05, and tendencies were discussed at 0.05 < *P* ≤ 0.10.

Yolk fatty acid composition and vitamins A and E were analyzed from composite samples prepared at the genotype × time level. Because these samples were pooled and did not provide analytical replication, these data were not subjected to inferential statistical analysis and are presented descriptively.

## Results

### Pasture utilization and botanical selection

**Fig. 1:**
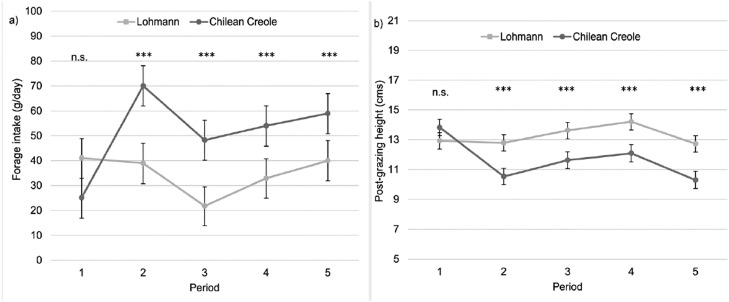
Genotype × period interaction for forage intake (a) and residual pasture height (b).

Forage intake differed between genotypes and varied over time, resulting in a significant genotype × period interaction (*P* < 0.01; Fig. 1a; Table 2). On average across periods, Chilean Creole hens consumed more pasture than Lohmann Brown hens (51.2 vs. 34.9 g/hen per day, respectively). Lohmann Brown hens showed relatively low and stable estimated forage intake over time, whereas Chilean Creole hens increased intake from period 1 to period 2 and maintained greater values than Lohmann Brown hens in subsequent periods. Post-grazing sward height also showed a significant genotype × period interaction (*P* < 0.05; Fig. 1b; Table 2). Residual sward height was similar between genotypes in period 1 but was higher in Lohmann Brown plots than in Chilean Creole plots from period 2 onwards. In contrast, pre-grazing height and pre-grazing botanical composition variables did not show significant genotype, period, or genotype × period effects (*P* > 0.10; Table 2). For post-grazing botanical composition, no genotype or genotype × period effects were detected, although grasses, legumes, and broad leaves varied among periods (*P* < 0.05; Table 2).

**Table 2:** Pasture height, botanical composition, and dry matter intake of Lohmann Brown and Chilean Creole laying hens.

Variable	Lohmann Brown	Chilean Creole	P1	P2	P3	P4	P5	SEM	Genotype	Period	Genotype × period
Pre-height (cm)	14.8	14.7	14.3	14.8	14.6	15.1	14.9	0.38	0.651	0.342	0.689
Post-height (cm)	13.3	11.7	13.4	11.7	12.6	13.1	11.5	0.55	0.0003	0.011	0.047
Forage intake (g/hen)	34.9	51.2	32.9	54.5	34.9	43.5	49.5	8.03	0.0004	0.007	0.007
Grasses-pre (%)	71	76	71	78	73	69	74	6.30	0.245	0.705	0.989
Grasses-post (%)	78	80	72	89	80	70	84	5.90	0.597	0.033	0.154
dGr (%)	7.21	4.36	1	11	7	0.64	10	6.56	0.517	0.438	0.337
Legumes-pre (%)	16	13	13	10	14	18	18	4.56	0.295	0.344	0.693
Legumes-post (%)	12	10	11	5	8	20	13	4.19	0.582	0.027	0.122
dLeg (%)	−4.11	−2.45	−2	−4.96	−6	2.4	−5.97	5.79	0.669	0.610	0.620
Broad leaves-pre (%)	13	12	16	12	14	13	8	3.96	0.514	0.353	0.814
Broad leaves-post (%)	10	10	18	7	12	10	3.6	4.17	0.843	0.046	0.630
dBL (%)	−3	−1.9	1	−6	−1	−3	−3.88	3.36	0.596	0.376	0.528

Abbreviations: P1-P5 = grazing periods; dGr = pre- to post-grazing difference for grasses; dLeg = pre- to post-grazing difference for legumes; dBL = pre- to post-grazing difference for broad leaves.

Species composition and its change with grazing differed among functional groups and varied across periods mainly in post-grazing measurements. Before grazing, grasses were the dominant component of the sward, averaging approximately 71 to 76% across genotypes, whereas legumes and broad leaves represented smaller proportions (Table 2). Pre-grazing botanical composition did not differ between genotypes and was not affected by period or genotype × period interaction (*P* > 0.10; Table 2). After grazing, grasses remained the dominant component, while legumes and broad leaves represented smaller proportions. Post-grazing botanical composition was affected by period for grasses, legumes, and broad leaves (*P* < 0.05), but no genotype or genotype × period effects were detected (Table 2). The pre- to post-grazing differences were generally positive for grasses and negative for legumes and broad leaves, indicating an overall decline in the relative contribution of legumes and broad leaves after grazing, although dLeg showed a positive numerical value in Period 4. Given the variability associated with botanical composition measurements, this isolated value was interpreted as a numerical fluctuation rather than as evidence of a consistent increase in post-grazing legume proportion. These results suggest selective use of legumes and broadleaf species by hens, with no evidence of genotype-specific botanical selection.

### Egg production and egg quality

Egg production and egg weight did not differ between genotypes during the recording period (*P* > 0.05; Table 3). However, both variables showed temporal variation, with egg weight differing across sampling periods (*P* = 0.042; Table 3). For external quality traits, LB eggs had greater fresh shell weight than ChC eggs (*P* < 0.001), whereas the proportion of shell was slightly higher in ChC eggs (*P* = 0.049; Table 3). Shell thickness was greater in ChC eggs than in LB eggs (*P* = 0.034; Table 3). Shell dirtiness scores were higher in ChC than in LB eggs (*P* < 0.001; Table 3). For internal traits, yolk color score was higher in ChC eggs than in LB eggs (*P* = 0.002; Table 3). ChC eggs also presented greater yolk height and yolk diameter (*P* ≤ 0.003; Table 3). Albumen height, Haugh units, and albumen index did not differ between genotypes (*P* > 0.05), although all these traits varied across sampling periods (*P* ≤ 0.003; Table 3).

**Table 3:** External and internal egg quality traits of Lohmann brown and chilean creole hens under free-range conditions.

Variable	Lohmann Brown	Chilean Creole	P0	P1	P2	P3	P4	P5	SEM	Genotype	Period	Genotype × period
Egg weight (g)	61.6	60.8	61.0	59.0	64.0	61.9	59.7	61.6	1.15	0.389	0.042	0.834
Egg yield (eggs/hen/ d)	0.48	0.54	0.37	0.45	0.55	0.59	0.55	0.54	0.09	0.094	0.006	0.242
Degree of dirtiness	1.44	1.89	1.67	1.45	1.70	1.73	1.75	1.68	0.24	0.0003	0.624	0.320
Egg length (mm)	57.9	57.7	57.6	57.4	58.8	58.0	57.5	57.9	0.52	0.573	0.296	0.863
Egg width (mm)	43.8	43.4	43.5	43.0	44.2	43.8	43.4	43.7	0.38	0.197	0.153	0.897
Shape index (%)	75.67	75.34	75.53	75.0	75.18	75.57	75.57	76.18	0.89	0.575	0.881	0.623
Fresh shell weight (g)	8.40	7.72	8.05	7.85	8.32	7.68	8.30	8.16	0.27	<0.0001	0.062	0.108
Dry shell weight (g)	5.84	5.99	5.81	5.82	6.29	5.96	5.68	5.94	0.17	0.210	0.102	0.653
Shell proportion (%)	9.48	9.86	9.55	9.86	9.86	9.62	9.50	9.64	0.27	0.049	0.793	0.832
Shell thickness (mm)	0.48	0.50	0.52	0.50	0.48	0.47	0.48	0.5	0.01	0.034	0.1147	0.2819
Yolk color score	6.93	8.06	9.09	7.88	7.63	7.10	7.10	6.18	0.31	0.002	<0.001	0.418
Haugh units	79.9	79.9	77.7	78.3	83.7	72.9	84.3	82.6	1.95	0.969	0.002	0.302
Albumen height (mm)	6.68	6.59	6.34	6.28	7.33	5.68	7.18	6.99	0.28	0.684	0.003	0.260
Yolk height (mm)	16.6	17.3	17.0	16.7	17.9	15.9	16.9	17.1	0.21	0.003	<0.001	0.868
Yolk diameter (mm)	41.0	43.7	41.5	41.2	42.4	43.4	42.7	43.0	0.45	<0.001	0.012	0.985
Albumen diameter (mm)	91.6	88.6	93.2	90.9	86.5	96.4	87.0	86.7	1.58	0.040	0.001	0.617
Yolk index	0.41	0.40	0.41	0.41	0.42	0.37	0.40	0.40	0.01	0.040	<0.001	0.787
Albumen index	0.08	0.05	0.07	0.07	0.09	0.06	0.08	0.08	0.004	0.864	0.002	0.404

Abbreviations: P0-P5 = egg sampling periods. P0 = baseline sampling period; P1–P5 = grazing periods.

### Functional components: fatty acids and vitamins

Yolk fatty acid and vitamin data are presented descriptively because composite samples were used and results are shown in Table 4. After pasture access, eggs from both genotypes showed numerically higher polyunsaturated fatty acids and omega-3 fatty acids and a lower omega-6:omega-3 ratio than baseline samples. Vitamin A concentration also increased numerically after pasture access in both genotypes, whereas vitamin E decreased slightly across sampling points. Because these values were obtained from pooled samples, they should be interpreted as exploratory patterns associated with pasture access rather than as statistically tested genotype or time effects.

**Table 4:** Descriptive fatty acid composition and vitamin content of egg yolk before and after grazing in Lohmann Brown and Chilean Creole laying hens.

Variable	Lohmann Brown Pre-grazing	Lohmann Brown Post-grazing	Chilean Creole Pre-grazing	Chilean Creole Post-grazing
*Fatty acids (%)*				
SFA	34.30	34.17	36.41	35.71
MUFA	42.17	38.66	40.66	37.86
PUFA	10.80	14.58	9.14	15.58
Omega-6	9.98	13.22	8.18	13.70
Omega-3	0.81	1.36	0.95	1.89
Omega-6:omega-3 ratio	12.28	9.72	8.58	7.26
*Vitamins*				
Vitamin A (µg RE)	245	292	165	197
Vitamin E (mg)	1.50	1.10	1.50	1.00

Data are descriptive because yolk samples were pooled at the treatment level. Post-grazing values represent the final sampling point after pasture access.

### Behavioral time budgets

The proportion of hens, from each genotype, that displayed each behavior are presented in Table 5. Most behavioral traits were influenced by sampling period rather than genotype, indicating that behavioral variation was primarily driven by temporal adaptation to the free-range environment. Sleeping behavior decreased across periods, and a significant genotype × period interaction was detected (*P* < 0.05), indicating temporal differences in resting patterns between Lohmann Brown and Chilean Creole hens. Preening behavior increased over time (*P* < 0.01), whereas feather pecking decreased across periods (*P* < 0.05), suggesting progressive adaptation to the outdoor system with reduced expression of potentially undesirable behaviors. Walking and confrontation behaviors also varied across periods (*P* < 0.05), but no main genotype effects were detected (*P* > 0.05). Feeding-related behaviors showed strong temporal effects: eating behavior differed across periods (*P* < 0.001), and foraging behavior increased over time (*P* < 0.01). Genotype significantly affected foraging (*P* < 0.05) during periods 1 and 2, where more Chilean Creole hens displayed foraging behavior (*P* = 0.049 during P1 and *P* = 0.006 at P2). Drinking behavior was also influenced by period (*P* < 0.05), with no genotype effect (*P* > 0.05).

**Table 5:** Proportion Lohmann brown and Chilean creole hens displaying the observed behaviors under free-range conditions.

Category	Behavior	Lohmann Brown	Chilean Creole	SEM	Genotype	Period	Genotype × period
Static	Sleeping	3.4	1.6	4.4	0.170	0.005	0.022
Static	Preening	15.0	20.0	12.2	0.172	0.002	0.696
Static	Feather pecking	4.8	7.4	5.6	0.112	0.011	0.066
Static	Head scratching	6.6	4.4	8.4	0.364	0.051	0.897
Dynamic	Walking	28.8	26.2	15.8	0.554	0.032	0.468
Dynamic	Confrontation	2.6	5.2	13.3	0.488	0.009	0.736
Feeding	Eating	29.6	32.0	12.1	0.526	<0.001	0.189
Feeding	Foraging	25.2	32.4	14.8	0.104	<0.001	0.137
Feeding	Drinking	19.4	20.4	17.2	0.861	0.011	0.998
Feeding	Feeder pecking	13.6	8.4	10.6	0.094	0.030	0.399

Values represent the mean percentage of birds observed performing each behavior per plot and observation day. Because values were averaged across repeated scans and observation days, percentages should be interpreted by behavior and are not intended to sum to 100 within genotype.

## Discussion

### Pasture utilization and botanical selection

In the present rotational free-range system, apparent pasture dry matter intake was greater in Chilean Creole hens than in Lohmann Brown hens, although this response varied over time as indicated by the genotype × period interaction. Under standardized concentrate supplementation, voluntary herbage intake may become partially constrained or buffered, reducing detectable genotype differences in total pasture intake, as reported in free-range and pasture-access systems (Mugnai et al., 2014; Singh and Cowieson, 2013).

However, genotype-related differences in pasture use have been reported under Chilean free-range conditions. Gandarillas et al. (2025) observed greater pasture consumption in Isa Brown hens compared with White Leghorn, suggesting that genetic background can influence herbage intake under certain management and pasture conditions. Differences among studies may therefore depend not only on genotype but also on pasture availability, concentrate allowance, and genotype-related traits such as body size and activity patterns.

Although apparent pasture intake was greater in Chilean Creole hens than in Lohmann Brown hens, changes in botanical composition between pre- and post-grazing samples revealed selective consumption of legumes and broadleaf species. Therefore, pasture use in this study should be interpreted through both total estimated dry matter intake and botanical selection. Free-range hens are known to preferentially consume nutrient-dense plant fractions rather than grasses alone (Horsted et al., 2007). In polyphytic swards such as those used in this experiment, legumes and certain broadleaf species may provide higher crude protein and bioactive compound concentrations than temperate grasses. Consequently, pasture effects should be interpreted not only through total dry matter intake but also through botanical selection and forage quality.

### Egg production and egg quality

Egg production values were lower than expected for commercial laying hybrids at this age. However, these results should be interpreted considering that hens were approximately 70 wk old and had been transferred to the experimental facilities from a floor-housing system, without previous experience in a rotational free-range system or direct pasture use. Thus, the transition to a novel outdoor environment, pasture exposure, rotational movement of plots, and the relatively short adaptation and evaluation period may have contributed to the reduced laying rate observed in both genotypes. No severe environmental stress event or systematic undercounting due to out-of-nest laying was observed during the study. Therefore, the low egg production values likely reflect the combined effects of late laying age, environmental transition, and adaptation to the rotational free-range system rather than a genotype-specific failure in productive performance.

Egg production and egg weight did not differ between genotypes, indicating that pasture access under controlled rotational management did not compromise the productivity of the commercial LB hens and did not confer a production advantage to ChC hens at 70 weeks of age. In alternative production systems, genotype effects on laying performance are often reduced when nutritional supply is adequate, as birds obtain most of their energy and nutrient requirements from the concentrate ration (; Mugnai et al., 2014).

Egg weight did not differ between genotypes, suggesting that both Chilean Creole and Lohmann Brown hens maintained comparable egg size under the rotational free-range conditions of this study. This response may reflect the standardized concentrate supplementation provided to all hens, which likely covered most nutritional requirements despite differences in apparent pasture intake. Egg weight is strongly influenced by genetic selection and body size, particularly at late stages of lay (Rakonjac et al., 2014). Therefore, the larger egg size observed in LB hens likely reflects commercial genetic potential rather than differences in pasture intake.

External shell traits differed between genotypes. Although LB eggs exhibited higher fresh shell weight, this variable should be interpreted cautiously because it may be influenced by residual moisture or albumen adhering to the shell before drying. In contrast, dry shell weight, shell proportion, and shell thickness provide more objective information on shell material deposition (Roberts, 2004). Dry shell weight did not differ between genotypes, whereas ChC eggs showed greater shell proportion and shell thickness, suggesting genotype-specific shell deposition patterns rather than differences in calcium intake, which was standardized across treatments.

Shell dirtiness scores were higher in ChC eggs. In free-range systems, shell contamination is commonly associated with environmental exposure and nest-use behavior rather than intrinsic shell quality (Sossidou and Elson, 2009; van Staaveren et al., 2019). These findings emphasize the importance of nest management and environmental design to minimize egg contamination in pasture-based production systems.

For internal quality traits, ChC eggs presented greater yolk pigmentation and larger yolk dimensions. Yolk color is largely influenced by dietary carotenoid intake and deposition efficiency (Karadas et al., 2006; Titcomb et al., 2019). Because apparent pasture intake was greater in Chilean Creole hens, the enhanced pigmentation in ChC eggs may reflect greater intake of carotenoid-rich plant material, selective ingestion of legumes and broadleaf species, or genotype-dependent deposition efficiency. Such differences are particularly relevant for market differentiation, as yolk color strongly influences consumer preferences (Berkhoff et al., 2020).

The descriptive increase in yolk omega-3 fatty acids and the lower omega-6:omega-3 ratio after pasture access are consistent with previous reports showing that outdoor access and forage intake can modify yolk lipid composition. However, the present results should be interpreted cautiously because yolk samples were pooled at the genotype × time level and therefore did not allow statistical testing. Consequently, these data support only an exploratory interpretation that pasture access may have contributed to changes in yolk functional components in both genotypes. Future studies should include replicated yolk samples at the plot level to determine whether these changes are statistically associated with genotype, time, or their interaction.

### Behavioral patterns under free-range conditions

Behavioral observations indicated temporal adaptation of hens to the free-range environment. Across sampling periods, resting and feather-pecking behaviors decreased, whereas preening and foraging increased. Feather pecking is frequently associated with environmental stress or social instability (Lay et al., 2011; Rodenburg et al., 2013; Relić et al., 2019); therefore, its reduction over time suggests improved acclimation to the outdoor system.

Access to outdoor areas allows hens to express natural behaviors such as foraging, scratching, and exploratory pecking (Chielo et al., 2016; Sossidou and Elson, 2009). Previous studies have reported that enriched outdoor environments can increase range use and reduce behavioral problems (Nagle and Glatz, 2012). The behavioral stabilization observed during the present trial suggests successful adaptation of both genotypes to the rotational free-range conditions.

Overall, genotype differences in behavioral patterns were limited, indicating that both commercial and local hens were capable of adjusting to the management system when environmental conditions were appropriate.

## Conclusions

Collectively, these findings indicate that Chilean Creole hens exhibited greater apparent pasture consumption than Lohmann Brown hens, but this did not translate into greater egg production under the rotational free-range management conditions of this study.

Nevertheless, genotype influenced selected egg quality traits, particularly yolk pigmentation and shell characteristics. These results suggest that Chilean Creole hens may represent a useful local genetic resource for pasture-based egg production systems, providing differentiated egg quality attributes without compromising laying performance.

## Funding

This research was funded by the Vicerrectoría de Investigación, Desarrollo y Creación Artística (VIDCA) of the Universidad Austral de Chile, through the project “Production of functional eggs from hens with high foraging capacity”, under the 2023 Concurso Proyectos de Investigación Aplicada e Innovación.

## Disclosures

All authors declare no conflict of interest.
